# Instrumented ligamentotaxis and stabilization of compression and burst fractures of dorsolumbar and mid-lumbar spines

**DOI:** 10.4103/0019-5413.36999

**Published:** 2007

**Authors:** Myung-Sang Moon, Won-Tae Choi, Doo-Hoon Sun, Jong-Woo Chae, Jong-Seon Ryu, Han Chang, Jin-Fu Lin

**Affiliations:** Department of Orthopedic Surgery, Sun General Hospital, Daejeon, Korea; * Department of Spine Surgery, Taipei Hospital, D.O.H, Taipei, Taiwan

**Keywords:** Burst fracture, compression fracture, dorsolumbar and lumbar, fracture, short segment fixation, spine

## Abstract

**Background:**

Controversy continues regarding the best treatment for compression and burst fractures. The axial distraction reduction utilizing the technique employing the long straight rod or curved short rod without derotation to reduce fracture are practised together with short segment posterolateral fusion (PLF). Effects of the early postoperative mobilization without posterolateral fusion on reduction maintenance and fracture consolidation were not evaluated so far. The present prospective study is designed to assess the effectiveness of i) reduction and restoration of sagittal alignment, ii) no posterolateral fusion on the reduced, fractured vertebral body and injured disc, iii) fracture consolidation and iv) the fate of the unfused cephalad and caudal injured motion segments of the fractured vertebra.

**Materials and Methods:**

The study includes 15 Denis burst and two Denis type D compression fractures between T_12_ and L_3_. The lordotic distraction technique was used for ligamentotaxis utilizing the contoured short rods and pedicle screw fixator. Three vertebrae including the fractured one were fixed. The patients after surgery were braced for ten weeks with activity restriction for 2-4 weeks. The patients were evaluated for change in vertebral body height, sagittal curve, reduction of retropulsion, improvement in neural deficit. The unfused motion segments, residual postoperative pain and bone and metal failure were also evaluated.

**Results:**

The preoperative and postreduction percentile vertebral heights at, zero (immediate postoperative), at three, six and 12 months followup were 62.4, 94.8, 94.6, 94.5 and 94.5%, respectively. The percentages of the intracanal fragment retropulsion at preoperative, and postoperative at zero, 3, 6 and 12 months followup were 59.0, 36.2,, 36.0, 32.3, and 13.6% respectively.

The preoperative and postreduction percentile loss of the canal dimension and at zero, three, six and 12 months were 52.1, 45.0, 44.0, 41.0 and 29% respectively suggesting that the under-reduced fragment was being resorbed gradually by a remodeling process. The mean initial kyphosis of 33° became mean 2° immediately after reduction and mean 3° at the final followup. The fractured vertebral bodies consolidated in an average period of ten weeks (range 8-14 weeks). The restored disc heights were relatively well maintained throughout the observation period. All paraparetic patients recovered neurologically. There were no postoperative complications.

**Conclusion:**

Instrument-aided ligamentotaxis for compression and burst fractures utilizing the short contoured rod derotation technique and the instrumented stabilization of the fractured spine are found to be effective procedures which contribute to the fractured vertebral body consolidation without recollapse and maintain the motion segment function.

Compression and burst fractures of dorsolumbar and mid-lumbar spines can be treated conservatively^1^–^13^ based on the severity and stability of fractures. The anterior or posterior surgery and short or long instrumentation^7^–^14^ are still controversial issues. Long segment instrumentation was preferred as it provides more stable fracture fixation to prevent vertebral body collapse and allows the early mobilization as compared to short segment instrumentation. Intact ligaments are essential for short or long posterior instrumented ligamentotaxis and stabilization of these fractures.

An intact anterior longitudinal ligament (ALL) prevents the occurrence of over-distraction of the fractured vertebra. A retropulsed fragment with a ligamentous attachment in the spinal canal can be reduced to a certain degree by ligamentotaxis. Retropulsed fragments near the midline are pulled back into place partly by the 0.5-1 cm-wide superficial fibers of the posterior longitudinal ligament (PLL) and fragments lying more laterally are reduced by their attachment to the 1 cm-wide segmental deep layer of PLL.^15^

The present prospective study was conducted on thoracolumbar and lumbar compression and burst fractures treated by short segment posterior instrumented (SSPI) reduction without posterolateral fusion to assess the effectiveness of i) reduction and restoration of sagittal alignment, ii) nonposterolateral fusion on the reduced, fractured vertebral body and injured disc, iii) fracture consolidation and iv) the fate of the unfused cephalad and caudal injured motion segments of the fractured vertebra. Hardware problems and effects of the postoperative restriction of the patient's activity and bracing on fracture healing were evaluated^7^,^16^ although there were no controls in this study.

## MATERIALS AND METHODS

Seventeen patients with 19 vertebral fractures with intact posterior-ligamento-osseus complex injuries constituted the clinical material for this study. Fractures were confined to the lower thoracic and lumbar regions (T11 to L3). Denis type compression fractures were found in two patients and Denis burst fractures in 15 patients. Six patients had incomplete paralysis on admission [Table 1]. Frankel's grade was used in neurological assessment. The fractures were examined by plain X-ray, computed tomography (CT) and magnetic resonance imaging (MRI).

**Table 1:** Levels of fractures and status of neurology *n* = 17, levels: 19

Levels	Nonparalytics	Incomplete paralytics	Total	Remarks
T_11_	1	1	2	Frankel's D
T_12_	2*	2	4*	Frankel's C
L_1_	4	2	6	Frankel's D
L_2_	2	1	3	Frankel's D
L_3_	4*	0	4*	Frankel's D
Total	13*	6	19*	

* Two patients had two level fractures (T_12_, L_3_); six had Frankel's type 1 incomplete paralysis

The short segment instrumentation procedure was performed for a) compression fracture > 30° kyphosis and 40% anterior column collapse, b) burst fracture > 25° kyphosis and > 50% vertebral height loss, > 40% canal compromise and c) unstable burst fracture with neurologic deficit. The rod derotation technique with the short contoured rods based on the rod sleeve fracture reduction principle was utilized for fracture reduction.^7^,^17^

**Figure 1:**
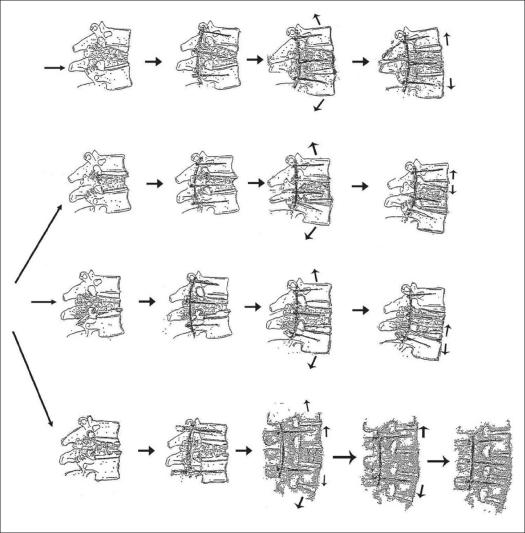
A technique of instrumented reduction of compression and burst fractures: Short vertebral fixation construct is used: three vertebral body fixations: one cephalad vertebra (ICV) + fractured vertebra (FV) + one caudate vertebra (ICV) construct. Arrows indicate the direction of distraction and the motion segment in which distraction force is applied. Posterior instrumentation with contoured rods, which provide consistent anatomic and lordotic distraction loads across the longitudinal axis and disc space, should best correct the vertebral height and intracanal fragment, while straight rods only provide axial distraction load. The uppermost column illustrates the lordotic distraction technique for compression fracture utilizing the contoured rods. This technique provides consistent anatomic and lordotic distraction loads across the longitudinal axis and uninjured ligament around the disc space should best correct the retropulsed vertebral height and intracanal fragment. Lower three columns illustrate the three reduction steps for instrumented ligamentotaxic reduction of superior (Denis type B) or inferior half body (Denis type C) burst fractures. The lowest column (Denis type burst A) illustrates the four steps of the reduction procedure, utilizing both upper and lower annular ligaments of the fractured vertebra

Short vertebral fixation construct was used with three vertebral body fixations: one cephalad vertebra + fractured vertebra (FV) + one caudad vertebra construct. The reduction techniques employed by the authors are illustrated in Figure 1. The uppermost column [Figure 1] illustrates the lordotic distraction technique for pure compression fractures utilizing the contoured rods. This technique provides consistent anatomic and lordotic distraction loads across the longitudinal axis and the ligament around the disc space should best correct the vertebral height and intracanal fragment. The lower three columns illustrate several steps of the procedure for three different types of burst fractures of the vertebral body. The upper two of the three columns illustrate the reduction steps for the burst fractures of the upper or lower half of the vertebral body. The lowest column illustrates the reduction steps for the severe burst fractures involving the entire vertebral body including the superior and inferior end-plates.

Postoperative management: The patients' activities were strictly restricted for 2-4 weeks and all patients were braced by a thoracolumbosacral orthoses (TLSO) over ten weeks.^18^ Patients with severely shattered burst fractures were confined to bed to prevent early recollapse and mobilized depending on radiographic evidence of fracture consolidation. Postoperative followup ranged from 12-40 months. Preoperative and postoperative measurements of several factors was performed at zero (immediate postoperative), three, six and 12 months and are listed below:

1. Accuracy of fracture reduction (percentile reduction)
2. Time of fracture consolidation
3. Changes in anterior and posterior vertebral body heights before fracture consolidation
4. Pre- and postoperative chronological changes of the intracanal fragment:
  - degree of posterior displacement of the retropulsed fragment
  - canal occupying ratio by the retropulsed fragment
5. Changes in the sagittal curve (kyphosis; Cobb's angle)

## RESULTS

The average percentile vertebral heights at the preoperative and post reduction at zero, three, six and 12 months were 62.4, 94.8, 94.6, 94.5 and 94.5% respectively of the normal. The anterior heights at preoperative and post reduction at zero, three, six and 12 months were 48.1, 92.4, 92.2, 92.0 and 92.0% respectively and posterior heights at pre- and post-reduction at zero, three, six and 12 months were 86.7, 97.1, 97.1, 97.0 and 97.0%, respectively [Table 2]. Near normal vertebral body height was restored and the reduction was maintained thereafter. Centrally depressed fracture fragments without attachment of annular ligament could not be reduced.

**Table 2:** Percentile vertebral height against normal height and reduction rates

	Preop.	Postop.			
		
		Immediate	3 months	6 months	12 months
Average % (middle height)	62.4%	94.8%	94.6%	94.5%	94.5%
(anterior height /posterior height)	(48.1/86.7)	(92.4/97.1)	(92.2/97.1)	(92.0/97.0)	(92.0/92.0)

Fracture consolidation as seen in radiograph took an average of ten weeks (8-14 weeks), which was proven by the postoperatively regained vertebral height changes. The percentile degrees of intracanal fragment retropulsion at preoperative and post reduction at zero, three, six and 12 months' followup were 59.0, 36.2, 36.0, 32.3 and 13.6%, respectively [Table 3]. The percentile losses of the canal dimension (area) at preoperative and post reduction at zero, three, six and 12 months' followup were 52.1, 45.0, 44.0, 41.0 and 29.0%, respectively [Table 3].

**Table 3:** Percentile displacement rates (degree) of retropulsed fragments and canal-occupying ratio in the canals

	**Displacement of retropulsed fragment**					**Canal occupying dimensional Rate of retropulsed fragment**				
		
	Preop	Postop				Preop	Postop			
				
		0M	3M	6M	12M		0M	3M	6M	12M
	59.0%	36.2%	36.0%	32.3%	13.6%	52.1%	45.0%	44.0%	41.0%	29.0%
Differences		22.8%	0.2%	3.7%	18.7%		7.1%	1.0%	3.0%	12.0%

This data indicates that the reduction of the retropulsed fragment by instrumentation was not complete and that the unreduced part of the retropulsed fragment could be resorbed gradually by remodelling. Thus, it appears that the degrees of the reduction of the collapsed vertebral height and retropulsed fragment did not match each other.

In all patients, trauma-induced deformity (initial kyphosis average = 33°) in the sagittal plane was corrected on surgery (residual kyphosis average = 2°) and maintained (residual kyphosis average = 3°) until the final followup. Thus, there were no clinically significant residual spinal deformities in any patient [Figures 2^3^]. Neurological recovery was observed in six patients wherein Frankel's C or D paralysis became Frankel's E. There were no hardware failures and screw toggles in the bones. No complications developed during the postoperative period in any patient. There was no residual pain or impairment in working capacity. Thus, overall outcomes were excellent with good adaptation to normal daily life.

**Figure 2.1:**
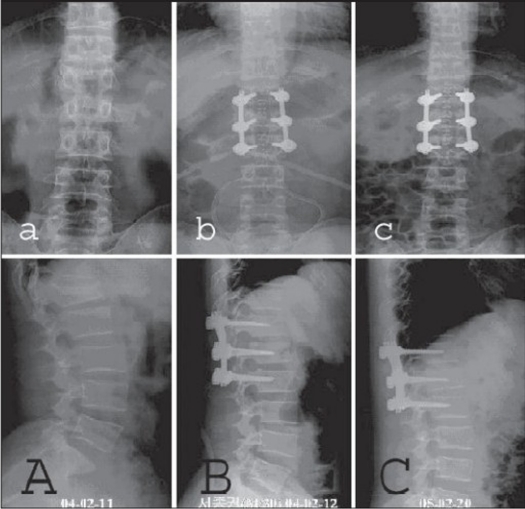
L_1_ burst fracture with retropulsed fragment: (aA) Preoperative plain roentgenograms show collapsed shattered upper 1/2 of L_1_ body with a retropulsed bony fragment and widened pedicle distance. (bB) Immediate postop roentgenograms show restored L_1_ vertebral height and sagittal alignment. (cC) 14 months followup shows fracture consolidated and restored basis height

**Figure 2.2:**
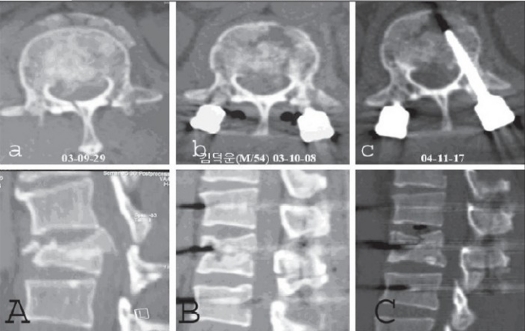
L_1_ burst fracture with retropulsed fragment: (aA) Preoperative CT shows vertically collapsed upper 1/2 of L_1_ body with a retropulsed fragment and (bB) well-reduced fracture is seen in postop CT. (cC) Fracture consolidated on 13 months followup

**Figure 3.1:**
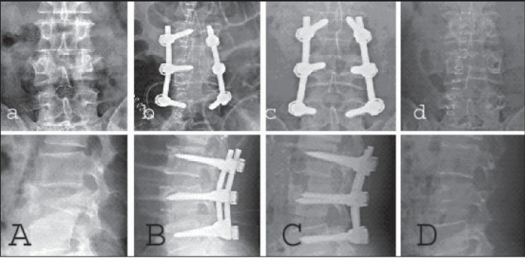
AP & Lateral x-ray showing L_3_ burst fracture: Preoperative (A) and postoperative at 0 (B), 12 months (C), 13 months after implant removal (D)

**Figure 3.2:**
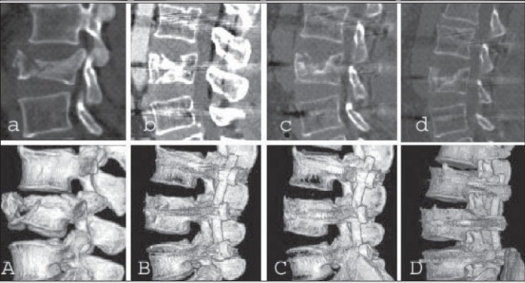
L_3_ burst fracture: Pre- (A) and postoperative (ten days, (B) eight (C) and 12 months (D)); serial CTs show the well-reduced fracture which has been well maintained until the last followup

**Figure 3.3:**
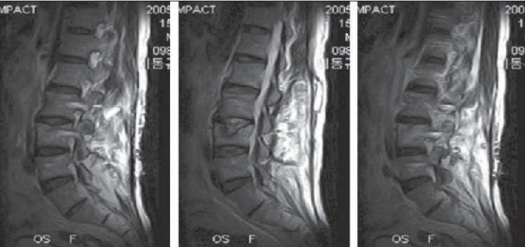
L_3_ burst fracture: MRI taken at postoperative 13 months, demonstrates the nondegenerated L_3-4_ disc with pseudo-Schmorl's node in the upper end-plate of L_4_

## DISCUSSION

The treatment of Denis' compression and burst fractures of the thoracolumbar and mid-lumbar spines still remains controversial. Good results were reported both in the nonoperative^7^,^18^–^20^ and operative treatment groups.^1^–^7^,^17^,^21^–^28^ Once the decision has been made to consider surgery, the goals must be clearly defined to aid the selection of the appropriate procedure to achieve optimal results. The major goals of the surgical treatment of the lower dorsal and lumbar spines are the restoration of normal spinal anatomy and neurological recovery.^5^,^18^ The efficiency of various surgical methods change, with the delay between injury and surgery. For old fractures which are over six weeks old, the mechanics of deformity correction are different because secondary changes have already occurred.^3^,^10^,^12^

Until now, most surgeons used the long instrumentation two or three levels cephalad and two levels caudad from the fractured vertebra for fracture fixation with short segment fusion construct^23^,^29^–^31^ to provide more secure fixation.^14^,^32^ The demerits of short instrumentation include a relatively high rate of proximal screw pullout and loss of correction in the setting of substantial anterior column compromise.^14^ Thus, short instrumentation has created much controversy because it seems to provide inadequate stabilization particularly in the presence of anterior column instability.^13^,^18^ This signifies the importance of restoring the anterior column stability to prevent future increase of kyphotic deformity after obtaining an initial correction. The insecure fixation of the fractured spine by short segment instrumentation as the main cause of fixation failure has been over-emphasized and long segment fixation and sacrifice of functioning 2-4 normal motion segments have been recommonded.^32^

The relationship between the reduction maneuver and fracture recollapse has rarely been discussed. Denis' type D compression fracture was easily reduced by rod rotation alone in most of the cases and the residual wedged body could be completely reduced by gentle additional distraction [Figure 1]. The iatrogenic anterior bony column defect should not be produced during the reduction maneuver by applying gentle distraction force, to prevent and/or minimize the corporal and disc recollapse and screw toggle or failure. We have been able to reduce the collapsed vertebral body height and restore of the normal sagittal alignment in burst fractures.

Injury to the anterior and middle columns may lead to a 70% decrease in support for the flexion load and a 70% decrease of torsional rigidity.^15^,^31^–^33^ Therefore, restoration of the anterior and middle columns is essential for maintaining the stability of the injured spine. The authors paid utmost attention to maintain the reduction till fracture consolidation and remobilize the immobilized motion segments after fracture healing. We hoped to obtain a similar satisfactory clinical outcome as is seen in conservatively treated minor trauma cases. In the current authors' series, good fracture reduction was observed, which could be maintained until fracture consolidation without fixation failure occurs.

For the treatment of compressed neural elements, indirect decompression technique by posterior instrumented ligamentotaxis, anterior-only approach to directly decompress the neural elements followed by internal fixation^7^,^13^,^18^ and combined anterior and posterior approaches have been suggested. Although a complete anatomic restitution of the spinal canal would seem to be desirable, no valid correlations have been established between the restoration of collapsed height and the reduction of the retropulsed fragment.

**Figure 4:**
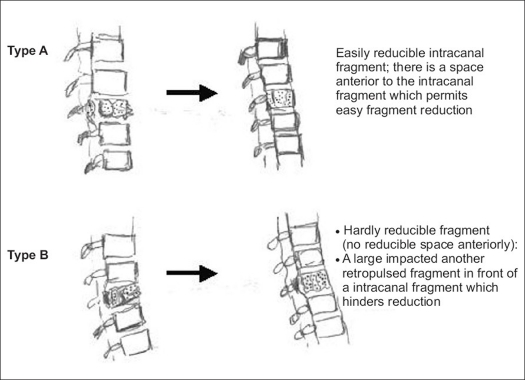
Line diagramme shows relationship of size of retropulsed fragment and/or other fracture fragments in front of the retropulsed fragment

The limitation of reduction of the retropulsed intracanal fragment by ligamentotaxis have not been discussed. The authors found two different situations in reduction of the intracanal fragment [Figure 4]. In type A, there is some space between the posterior wall of the fractured vertebra and the retropulsed fragment, while in type B, there is no space anteriorly for the reduction of the retropulsed fragment. Accurate delineation of the canal size is essential to correlate canal dimension and neurology in acute trauma cases.^34^,^35^ We measured the degree of posterior displacement of the retropulsed fragment and the canal occupying ratio of the fragment. The permissible degree of acute cord compression by the retropulsed fragment has been difined and it suggested 67% of retropulsion was the demarcation to cause neural damage in acute trauma cases.^36^,^37^

The efficacy of indirect decompression was greater when surgery was performed as early as possible, particularly within three days after the trauma. We could not correlate degree of the reduction of the retropulsed fragment with the height reduction.

Panjabi *et al.* determined the ideal reduction maneuvers as either a combination of 5 mm of distraction with 6° of extension or simply 10 mm of distraction to decompress the spinal canal and intervertebral foramina in a cadaveric L_1_ burst model.^15^,^37^,^38^ The combination of 5 mm of distraction with 6° of extension was adopted by using a straight rod with a sleeve, while 10 mm of distraction was employed by using straight rods. However, it was practically difficult to follow in the operating theatre because we used contoured rods. The tendency towards greater canal clearance after stabilization was apparent in our series although not significant clinically. Fracture consolidation took place relatively early in all patients, which was indicated indirectly by the arrest of height recollapse, maintenance of the restored sagittal alignment and maintenance of fixation.

We experienced that PLF was not helpful in maintaining the restored vertebral height and the sagittal alignment by the time of fracture consolidation.^18^ This was attributed to the vertebral body recollapse usually occurring before the establishment of the PLF and fracture consolidation. Also, the overall outcomes did not correlate with the establishment of PLF, while they did correlate with the consolidation of the fractured vertebral bodies.^18^,^39^ We attempted to preserve all motion segments if possible, because the patients' lumbar spine stiffened by fusion prevents them from enjoying the lifestyle and religions practices of the Far East.

Postoperative management was considered as one of the most important factors which contribute to the maintenance of the reduction until fracture consolidation and to the neural recovery. Early postoperative patients' mobilization has been listed as one of the merits of instrumentation surgery.^7^ However, the authors differ in their opinions regarding postoperative management of severe burst fractures, which are associated with anterior column instability. Therefore, an early postoperative patient's mobilization protocol was replaced by a gentle gradual mobilization protocol in the current series. Restriction of postoperative patients' activity for a certain period of time seems to contribute to earlier fracture consolidation with minimal recollapse of the regained body and disc heights although this regimen may raise some controversy. The authors conceived this idea based on their clinical experiences with poly-trauma patients' complicated with spine fracture,^17^ in whom there were no vertebral body recollapse after instrumented reduction and stabilization.^18^

There is no acceptable regimen for the removal of the implants. The removal of implants 12-16 months after surgery in patients where no posterolateral fusion has been performed has been recommonded.^39^,^40^ We removed the implants 9-12 months postoperatively after definite confirmation of fracture consolidation.^18^,^40^,^41^ Thus, the immobilized joints could be preserved by the time of implant removal.

Short term survey, lack of comparative studies between early and delayed postoperative patients' mobilization for i) reduction maintenance, ii) fracture consolidation, iii) between bone quality and fixation failure, iv) short and longer hospitalization on fracture reduction maintenance and consolidation can be listed as the limitations of this study. Further studies are planned to address these issues.

## CONCLUSION

It is concluded that short-segment posterior pedicle fixation with contoured rods for Denis' compression and burst fractures is an effective reduction and stabilization method and that nonfusion of the instrumented segment is recommended. Protected gradual postoperative patients' mobilization is thought to be a cost-effective management protocol in reducing fixation failure and for overall successful recovery.

## References

[1] Boucher HH (1997). Method for spinal fusion. Clin Orthop Relat Res.

[2] Edwards CC, Levine AM (1986). Early rod-sleeve stabilization of the injured thoracic and lumbar spine. Orthop Clin North Am.

[3] Kuner EH, Kuner A, Schlickewei W, Mullaji AB (1994). Ligamentotaxis with an internal spinal fixator for thoracolumbar fractures. J Bone Joint Surg Br.

[4] Butler JS, Walsh A, O'Byrne J (2005). Functional outcome of the burst fractures of the first lumbar vertebra managed surgically and conservatively. Int Orthop.

[5] Butt MF, Farooq M, Mir B, Dahr AS, Hussain A, Mumtaz M (2007). Management of unstable thoracolumbar spinal injuries by posterior short-segment spinal fixation. Int Orthop.

[6] Chang HG, Kim YW, Lee YB (2004). A prospective study of posterior instrumentation without fusion for the stable thoracolumbar fracture: Abstract book of 14th.

[7] Kaneda K, Taneichi H, Abumi K, Hashimoto T, Satob S, Fajiya M (1997). Anterior decompression and stabilization with the kaneda device for thoracolumbar burst fractures associated with neurological deficits. J Bone Joint Surg Am.

[8] Kostuik JP (1988). Anterior fixation for burst fractures of the thoracic and lumbar spine with or without neurological involvement. Spine.

[9] Kuklo TR, Polly DW, Owens BD, Zeidman SM, Chang AS, Klemme WR (2001). Measurement of thoracic and lumbar fracture kyphosis. Spine.

[10] Knope, Fabian HF, Bastian L, Blauth M (2001). Late results of thoracolumbar fractures after posterior instrumentation and transpedicular bone grafting. Spine.

[11] Louis CA, Gauthier VY, Louis RP (1998). Posterior approach wth Louis plates for fractures of the thoracolumbar and lumbar spine with and without neurologic deficits. Spine.

[12] Mikles MR, Stchur RP, Graziano GP (2004). Posterior instrumentation for thoracolumbar fractures. J Am Acad Orthop Surg.

[13] Moon MS, Kim I, Woo YK, Lee JJ (1981). Anterior interbody fusion in fracture and fracture-dislocation of the spine. Int Orthop.

[14] Korovessis P, Baikousis A, Zacparatos S, Petsinis G, Koureas G, Iliopoulos P (2006). Combined anterior plus posterior stabilization versus posterior short segment instrumentation and fusion for mid-lumbar (L2-L4) burst fractures. Spine.

[15] Panjabi MM, Oxland TR, Kifune M, Arand M, Wen L, Chen A (1995). Validity of the three-column theory of thoracolumbar fracture: A biomechanic investigation. Spine.

[16] Kim YM, Kim DS, Choi ES, Sohn HC, Park KJ, Jeong KI (2005). Results of non-fusion method in thoracolumbar and lumbar spinal fractures. J Korea Spine Soc.

[17] MeLain R, Benson DR (1999). Urgent surgical stabilization of spinal fractures in polytrauma patients. Spine.

[18] Moon MS, Choi WT, Moon YW, Kim YS, Moon JL (2003). Stabilization of fractured thoracic and lumbar spine with Cotrel-Dubousset instrument. J Orthop Surg (Hong Kong).

[19] Tropiano P, Huang RC, Louis CA, Poitout DG, Louis RP (2003). Functional and radiographic outcome of thoracolumbar and lumbar burst fractures managed by closed orthopedic reduction and casting. Spine.

[20] Willen J, Lindahl S, Nordwall A (1985). Unstable thoracolumbar fractures: A comparative clinical study of conservative treatment and Harrington instrumentation. Spine.

[21] Fontijne WP, de Klerk LW, Braakman R, Stijnen T, Tanghe HL, Steenbeek R (1992). CT scan prediction of neurological deficit in thoracolumbar burst fractures. J Bone Joint Surg Br.

[22] Gaines RW (2000). The use of pedicle screw internal fixation for the operative treatment of spinal disorders. J Bone Joint Surg Am.

[23] Gertzbein SD, Crowe PJ, Schwartz M, Rowed D (1992). Canal clearance in burst fractures using the AO internal fixator. Spine.

[24] Hardaker WT, Cook WA, Friedman AH, Fitch RD (1992). Bilateral transpedicular decompression and Harrington rod stabilization in the management of severe thoracolumbar burst fractures. Spine.

[25] de Peretti F, Cambas PM, Puch JM, Nasr ZG, Lovet J, Argenson C (1994). Modular construction (2 HS-1 SH), using Cotrel-Dubousset's universal instrumentation for comminuted fractures of the thoracolumbar junction. Comparison with various other constructions. Rev Chir Orthop Reparatrice Appar Mot.

[26] Ruan DK, Shen GB, Chui HX (1998). Shen instrumentation for the management of unstable thoracolumbar fractures. Spine.

[27] Shen WJ, Liu TJ, Shen YS (2001). Non-operative treatment versus posterior fixation for the thoracolumbar junction burst fractures without neurologic deficit. Spine.

[28] Starr JK, Hanley EN (1992). Junctional burst fractures. Spine.

[29] Chang KW (1992). A reduction-fixation system for unstable thoracolumbar burst fractures. Spine.

[30] Katonis PG, Kontakis GM, Loupasis GA, Aligizakis AC, Christoforakis JI, Velivassakis EG (1999). Treatment of unstable thoracolumbar and lumbar spine injuries using Cotrel - Dubusset instrumentation. Spine.

[31] Kifune M, Panjabi MM, Arand M, Liu W (1995). Fracture pattern and instability of thoracolumbar injuries. Eur Spine J.

[32] Parker JW, Lane JR, Karaikovic EE, Gaines RW (2000). Successful short segment instrumentation and fusion for thoracolumbar fractures: A consecutive 4½ - year series. Spine.

[33] Vaccaro AR, Kim DH, Brodke DS, Harris M, Chapman J, Schildhauer T (2004). Diagnosis and management of thoracolumbar spine fractures. Instr Course Lect.

[34] Schuman WP, Ragers JV, Sickler ME, Hanson JA, Crutcher JP, King HA (1985). Thoracolumbar burst fractures: CT dimensions of the spinal canal relative to postsurgical improvement. AJR Am J Roentgenol.

[35] Sjöström L, Karlström G, Pech P, Rauschning W (1996). Indirect spinal canal decompression in burst fractures treated with pedicle screw instrumentation. Spine.

[36] Sasso RC, Renkens K, Hanson D, Reilly T, McGuire RA, Best NM (2006). Unstable thoracolumbar fractures: Anterior-only versus short-segment posterior fixation. J Spinal Disord Tech.

[37] Spivak JM, Vaccaro AR, Cotler JM (1995). Thoracolumbar spine trauma II: Principles of management. J Am Acad Orthop Surg.

[38] Yue JJ, Sossan A, Selgrath C, Deutsch LS, Wilkens K, Testaiuti M (2002). The treatment of unstable thoracic spine fractures with transpedicular screw instrumentation: A 3 year consecutive series. Spine.

[39] Stromsoe K, Hem ES, Aunan E (1997). Unstable vertebral fractures in the lower third of the spine treated with closed reduction and transpedicular posterior fixation: A retrospective analysis of 82 fractures in 78 patients. Eur Spine J.

[40] Oner FC, Van Gils AP, Faber JA, Dhert WJ, Verbout AJ (2002). Some complications of common treatment schemes of thoracolumbar spine fractures can be predicted with magnetic resonance imaging: Prospective study of 53 patients with 71 fractures. Spine.

[41] Lee CS, Chung SS, Jung HW, Kim ES (2001). Decision of posterior fixation level by load-shearing classification in thoracolumbar and lumbar burst fractures (in Korean). J Korea Spine Surg.

